# (*E*)-4-Amino-*N*′-(2-nitro­benzyl­idene)benzohydrazide

**DOI:** 10.1107/S1600536812020855

**Published:** 2012-05-16

**Authors:** Zhong-Feng Shi, Jia-Ming Li

**Affiliations:** a College of Chemistry and Chemical Engineering, Qinzhou University, Qinzhou, Guangxi 535000, People’s Republic of China; b Guangxi Key Laboratory of Petrochemical Resource Processing and Process Intensification Technology, Guangxi University, Nanning, Guangxi 530004, People’s Republic of China

## Abstract

The title Schiff base compound, C_14_H_12_N_4_O_3_, displays an *E* conformation with respect to the C=N double bond [1.268 (3) Å]. The dihedral angle between the benzene rings is 3.2 (5)°, consistent with an essentially planar mol­ecule. In the crystal, N—H⋯O and N—H⋯N hydrogen bonds, as well as C—H⋯O inter­actions, link the mol­ecules into layers that stack along the *c* axis.

## Related literature

For the coordination chemistry of Schiff base and hydrazone compounds, see: Kucukguzel *et al.* (2006 ▶); Khattab *et al.* (2005 ▶); Karthikeyan *et al.* (2006 ▶). For a closely related 4-amino­benzohydrazide and its Schiff base structures and further background references, see: Xu (2012 ▶); Shi & Li (2012 ▶); Bakir & Green (2002 ▶).
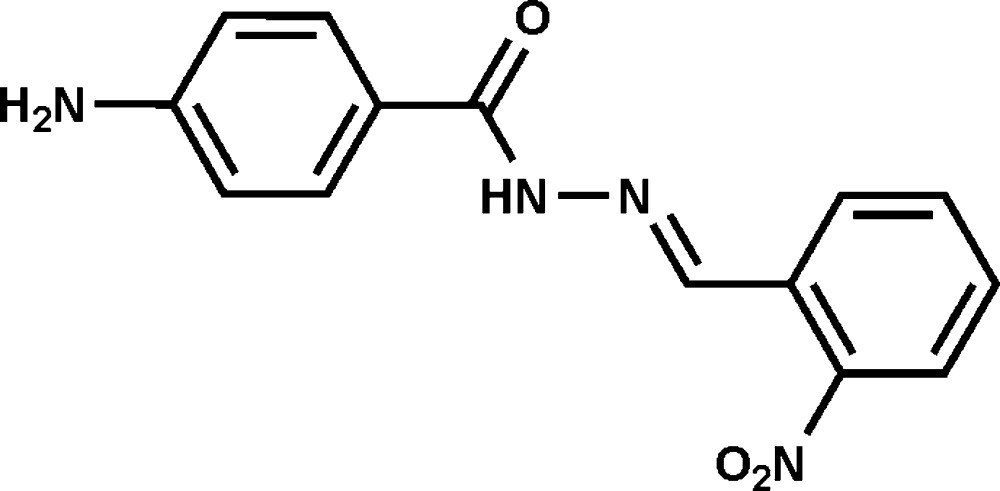


## Experimental

### 

#### Crystal data

C_14_H_12_N_4_O_3_
*M* *_r_* = 284.28
Monoclinic, 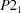

*a* = 6.4594 (13) Å
*b* = 4.5998 (13) Å
*c* = 20.598 (5) Å
β = 95.08 (4)°
*V* = 609.6 (3) Å^3^
*Z* = 2
Mo *K*α radiation
μ = 0.11 mm^−1^
*T* = 296 K
0.25 × 0.18 × 0.10 mm

#### Data collection

Bruker SMART CCD area-detector diffractometer
Absorption correction: multi-scan (*SADABS*; Sheldrick, 1996 ▶) *T*_min_ = 0.976, *T*_max_ = 0.989
4632 measured reflections
2710 independent reflections
1516 reflections with *I* > 2σ(*I*)
*R*_int_ = 0.048

#### Refinement

*R*[*F* ^2^ > 2σ(*F* ^2^)] = 0.045
*wR*(*F* ^2^) = 0.111
*S* = 1.05
2710 reflections
190 parameters
H-atom parameters constrained
Δρ_max_ = 0.27 e Å^−3^
Δρ_min_ = −0.22 e Å^−3^

### 

Data collection: *SMART* (Bruker, 2007 ▶); cell refinement: *SAINT* (Bruker, 2007 ▶); data reduction: *SAINT*; program(s) used to solve structure: *SHELXS97* (Sheldrick, 2008 ▶); program(s) used to refine structure: *SHELXL97* (Sheldrick, 2008 ▶); molecular graphics: *SHELXTL* (Sheldrick, 2008 ▶); software used to prepare material for publication: *SHELXTL*.

## Supplementary Material

Crystal structure: contains datablock(s) global, I. DOI: 10.1107/S1600536812020855/tk5092sup1.cif

Structure factors: contains datablock(s) I. DOI: 10.1107/S1600536812020855/tk5092Isup2.hkl

Supplementary material file. DOI: 10.1107/S1600536812020855/tk5092Isup3.cml

Additional supplementary materials:  crystallographic information; 3D view; checkCIF report

## Figures and Tables

**Table 1:** Hydrogen-bond geometry (Å, °)

*D*—H⋯*A*	*D*—H	H⋯*A*	*D*⋯*A*	*D*—H⋯*A*
N1—H1*A*⋯N1^i^	0.89	2.44	3.287 (4)	162
N1—H1*B*⋯O1^ii^	0.89	2.35	3.164 (3)	153
N2—H2⋯O1^iii^	0.86	2.13	2.843 (3)	142
C2—H2*A*⋯O1^ii^	0.93	2.56	3.329 (3)	140

Symmetry codes: (i) 
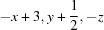
; (ii) 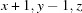; (iii) 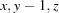.

**Fig. 1.**
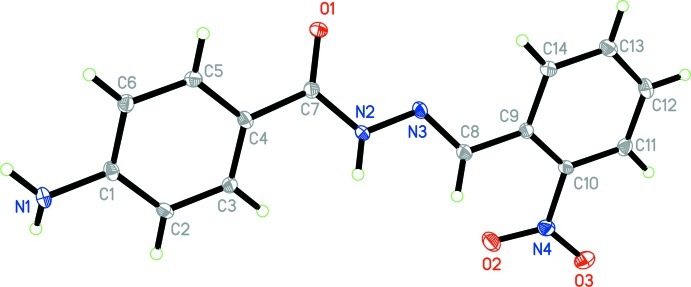
The molecular structure of the title compound, with displacement ellipsoids at the 30% probability level.

**Fig. 2.**
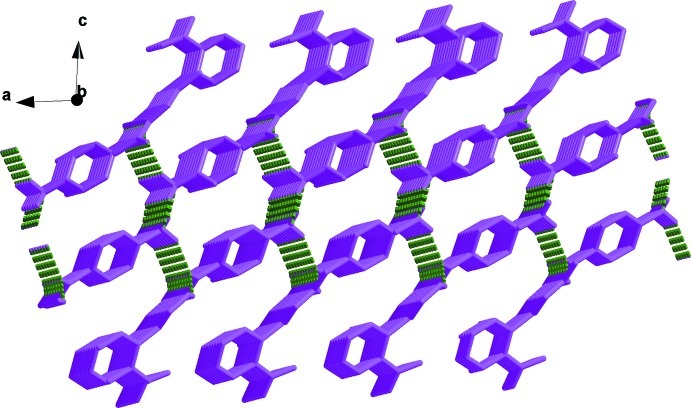
Crystal packing in the title compound where molecules are linked via N—H···O and N–H···N hydrogen bonds (dashed lines). Except for those involved in hydrogen-bonding interactions, H atoms have been omitted for clarity.

## supplementary crystallographic information

### Comment

Schiff bases are one of the most prevalent mixed-donor ligands in the field of coordination chemistry. They play an important role in the development of coordination chemistry related to catalysis and enzymatic reactions, magnetism, and supramolecular architectures (Karthikeyan *et al.*, 2006; Khattab, 2005; Kucukguzel *et al.*, 2006). Structures of Schiff bases derived from substituted 4-aminobenzohydrazide and closely related to the title compound have been reported earlier (Xu, 2012; Shi & Li, 2012; Bakir & Green, 2002). In order to explore new anti-bacterial compounds, a new hydrazone derivative was prepared and characterized crystallographically.

As shown in Fig. 1, the asymmetric unit of the title compound, (I), contains one independent molecule displaying an *E* configuration with respect to its C═N double bond. The dihedral angle between the two benzene rings is 3.2 (5)°. The bond lengths and angles are as expected for a compound of this type and agree with the other ligands belonging to the hydrazone series. The C8═N3 and C7═O1 bond lengths of 1.268 (3) and 1.226 (3) Å, respectively, are the expected values for such double bonds. In the crystal packing, it is noted that amino-H (H1A, H1B) and amide-H2A atoms are involved in forming intermolecular N—H···O and N—H···N hydrogen bonds (Fig. 2 and Table 1), linking the molecules into a two-dimensional layer structure that stacks along the *c* axis. Weak C—H···O interactions are also noted within the layer.

### Experimental

To a methanol solution (20 ml) of 2-nitrobenzaldehyde (1 mmol, 0.151 g) and 4-aminobenzohydrazide (1 mmol, 0.151 g), a few drops of acetic acid were added. The mixture was refluxed for 2 h and then cooled to room temperature to give a yellow solution. Crystals of the title compound were formed by gradual evaporation of the solvent over a period of 6 days at room temperature.

### Refinement

H-atoms were placed in calculated positions (C—H = 0.93 and N—H = 0.86–0.89 Å) and were included in the refinement in the riding model approximation, with *U*_iso_(H) set to 1.2*U*_eq_(C or N). In the absence of significant anomalous scattering effects, 1120 Friedel pairs were averaged in the final refinement.

### Crystal data

**Table:**

C_14_H_12_N_4_O_3_	*F*(000) = 296
*M**_r_* = 284.28	*D*_x_ = 1.549 Mg m^−^^3^
Monoclinic, *P*2_1_	Mo *K*α radiation, λ = 0.71073 Å
Hall symbol: P 2yb	Cell parameters from 7138 reflections
*a* = 6.4594 (13) Å	θ = 1.4–27.5°
*b* = 4.5998 (13) Å	µ = 0.11 mm^−^^1^
*c* = 20.598 (5) Å	*T* = 296 K
β = 95.08 (4)°	Block, yellow
*V* = 609.6 (3) Å^3^	0.25 × 0.18 × 0.10 mm
*Z* = 2	

### Data collection

**Table:**

Bruker SMART CCD area-detector diffractometer	2710 independent reflections
Radiation source: fine-focus sealed tube	1516 reflections with *I* > 2σ(*I*)
Graphite monochromator	*R*_int_ = 0.048
φ and ω scans	θ_max_ = 27.5°, θ_min_ = 2.0°
Absorption correction: multi-scan (*SADABS*; Sheldrick, 1996)	*h* = −8→7
*T*_min_ = 0.976, *T*_max_ = 0.989	*k* = −6→5
4632 measured reflections	*l* = −26→24

### Refinement

**Table:**

Refinement on *F*^2^	Primary atom site location: structure-invariant direct methods
Least-squares matrix: full	Secondary atom site location: difference Fourier map
*R*[*F*^2^ > 2σ(*F*^2^)] = 0.045	Hydrogen site location: inferred from neighbouring sites
*wR*(*F*^2^) = 0.111	H-atom parameters constrained
*S* = 1.05	*w* = 1/[σ^2^(*F*_o_^2^) + (0.0576*P*)^2^ + 0.2479*P*] where *P* = (*F*_o_^2^ + 2*F*_c_^2^)/3
2710 reflections	(Δ/σ)_max_ < 0.001
190 parameters	Δρ_max_ = 0.27 e Å^−^^3^
0 restraints	Δρ_min_ = −0.22 e Å^−^^3^

### Special details

**Table:**

Geometry. All esds (except the esd in the dihedral angle between two l.s. planes) are estimated using the full covariance matrix. The cell esds are taken into account individually in the estimation of esds in distances, angles and torsion angles; correlations between esds in cell parameters are only used when they are defined by crystal symmetry. An approximate (isotropic) treatment of cell esds is used for estimating esds involving l.s. planes.
Refinement. Refinement of F^2^ against ALL reflections. The weighted R-factor wR and goodness of fit S are based on F^2^, conventional R-factors R are based on F, with F set to zero for negative F^2^. The threshold expression of F^2^ > 2sigma(F^2^) is used only for calculating R-factors(gt) etc. and is not relevant to the choice of reflections for refinement. R-factors based on F^2^ are statistically about twice as large as those based on F, and R- factors based on ALL data will be even larger.

### Fractional atomic coordinates and isotropic or equivalent isotropic displacement parameters (Å^2^)

**Table:**

	*x*	*y*	*z*	*U*_iso_*/*U*_eq_	
O1	0.6908 (3)	1.3470 (4)	0.16727 (9)	0.0252 (4)	
O2	0.5696 (3)	0.5737 (5)	0.40854 (9)	0.0335 (5)	
O3	0.3389 (3)	0.4666 (5)	0.47354 (9)	0.0338 (5)	
N1	1.4552 (3)	0.7167 (6)	0.05402 (10)	0.0248 (5)	
H1A	1.5039	0.8252	0.0236	0.030*	
H1B	1.5583	0.6443	0.0811	0.030*	
N2	0.6727 (3)	0.9187 (5)	0.21889 (9)	0.0193 (4)	
H2	0.7097	0.7415	0.2215	0.023*	
N3	0.5305 (3)	1.0250 (5)	0.25824 (9)	0.0198 (4)	
N4	0.3968 (3)	0.6017 (5)	0.42806 (10)	0.0241 (5)	
C1	1.2814 (3)	0.8062 (6)	0.08357 (11)	0.0196 (5)	
C2	1.2309 (3)	0.6722 (6)	0.14030 (11)	0.0194 (5)	
H2A	1.3134	0.5234	0.1585	0.023*	
C3	1.0579 (3)	0.7606 (6)	0.16970 (11)	0.0190 (5)	
H3	1.0271	0.6715	0.2078	0.023*	
C4	0.9293 (3)	0.9809 (6)	0.14297 (11)	0.0188 (5)	
C5	0.9786 (3)	1.1101 (6)	0.08586 (11)	0.0200 (5)	
H5	0.8938	1.2555	0.0671	0.024*	
C6	1.1522 (3)	1.0262 (6)	0.05623 (11)	0.0213 (5)	
H6	1.1827	1.1162	0.0182	0.026*	
C7	0.7535 (3)	1.0973 (6)	0.17592 (11)	0.0189 (5)	
C8	0.4572 (3)	0.8400 (6)	0.29566 (11)	0.0196 (5)	
H8	0.5066	0.6525	0.2978	0.024*	
C9	0.2917 (3)	0.9327 (6)	0.33548 (11)	0.0188 (5)	
C10	0.2529 (3)	0.8118 (6)	0.39485 (11)	0.0194 (5)	
C11	0.0792 (4)	0.8859 (6)	0.42611 (11)	0.0230 (6)	
H11	0.0555	0.7975	0.4650	0.028*	
C12	−0.0568 (4)	1.0909 (7)	0.39888 (12)	0.0264 (6)	
H12	−0.1735	1.1405	0.4191	0.032*	
C13	−0.0189 (4)	1.2225 (6)	0.34132 (12)	0.0245 (5)	
H13	−0.1085	1.3647	0.3236	0.029*	
C14	0.1523 (3)	1.1439 (6)	0.30967 (12)	0.0218 (5)	
H14	0.1746	1.2331	0.2707	0.026*	

### Atomic displacement parameters (Å^2^)

**Table:**

	*U*^11^	*U*^22^	*U*^33^	*U*^12^	*U*^13^	*U*^23^
O1	0.0270 (9)	0.0148 (9)	0.0353 (10)	0.0046 (7)	0.0107 (7)	0.0022 (8)
O2	0.0270 (8)	0.0396 (13)	0.0347 (10)	0.0120 (9)	0.0078 (7)	0.0073 (10)
O3	0.0390 (10)	0.0295 (12)	0.0336 (10)	0.0006 (9)	0.0069 (8)	0.0107 (10)
N1	0.0206 (9)	0.0285 (13)	0.0269 (10)	0.0033 (10)	0.0103 (7)	−0.0009 (11)
N2	0.0187 (9)	0.0158 (10)	0.0247 (9)	0.0043 (8)	0.0083 (7)	0.0010 (9)
N3	0.0182 (8)	0.0198 (11)	0.0223 (9)	0.0026 (8)	0.0061 (7)	−0.0025 (9)
N4	0.0269 (10)	0.0202 (11)	0.0256 (10)	0.0020 (9)	0.0052 (8)	−0.0012 (10)
C1	0.0170 (10)	0.0202 (13)	0.0222 (10)	−0.0017 (10)	0.0047 (8)	−0.0073 (11)
C2	0.0182 (10)	0.0164 (13)	0.0236 (11)	0.0029 (9)	0.0021 (8)	−0.0020 (10)
C3	0.0193 (10)	0.0154 (13)	0.0232 (11)	−0.0012 (9)	0.0060 (8)	−0.0008 (10)
C4	0.0164 (9)	0.0189 (13)	0.0215 (10)	0.0004 (10)	0.0034 (7)	−0.0039 (10)
C5	0.0204 (10)	0.0170 (12)	0.0227 (11)	0.0012 (10)	0.0024 (8)	0.0011 (11)
C6	0.0232 (11)	0.0208 (13)	0.0208 (10)	−0.0012 (10)	0.0068 (8)	−0.0008 (10)
C7	0.0181 (10)	0.0171 (12)	0.0222 (11)	−0.0012 (10)	0.0044 (8)	−0.0026 (11)
C8	0.0188 (9)	0.0181 (12)	0.0223 (10)	0.0010 (10)	0.0038 (8)	−0.0014 (11)
C9	0.0157 (9)	0.0153 (12)	0.0258 (11)	−0.0009 (9)	0.0045 (8)	−0.0028 (10)
C10	0.0188 (10)	0.0153 (12)	0.0243 (11)	0.0014 (10)	0.0028 (8)	−0.0007 (11)
C11	0.0251 (11)	0.0230 (15)	0.0223 (11)	−0.0018 (11)	0.0094 (8)	−0.0004 (11)
C12	0.0203 (10)	0.0318 (16)	0.0283 (12)	0.0023 (11)	0.0084 (9)	−0.0031 (13)
C13	0.0223 (10)	0.0216 (14)	0.0293 (12)	0.0035 (11)	0.0020 (9)	−0.0028 (12)
C14	0.0215 (10)	0.0187 (14)	0.0254 (11)	0.0013 (10)	0.0035 (8)	0.0010 (11)

### Geometric parameters (Å, º)

**Table:**

O1—C7	1.226 (3)	C4—C5	1.380 (3)
O2—N4	1.226 (3)	C4—C7	1.474 (3)
O3—N4	1.211 (3)	C5—C6	1.378 (3)
N1—C1	1.386 (3)	C5—H5	0.9300
N1—H1A	0.8900	C6—H6	0.9300
N1—H1B	0.8900	C8—C9	1.467 (3)
N2—C7	1.346 (3)	C8—H8	0.9300
N2—N3	1.368 (3)	C9—C10	1.386 (3)
N2—H2	0.8600	C9—C14	1.397 (3)
N3—C8	1.268 (3)	C10—C11	1.385 (3)
N4—C10	1.470 (3)	C11—C12	1.378 (4)
C1—C2	1.385 (3)	C11—H11	0.9300
C1—C6	1.398 (3)	C12—C13	1.372 (4)
C2—C3	1.379 (3)	C12—H12	0.9300
C2—H2A	0.9300	C13—C14	1.381 (3)
C3—C4	1.393 (3)	C13—H13	0.9300
C3—H3	0.9300	C14—H14	0.9300
			
C1—N1—H1A	120.0	C5—C6—H6	119.9
C1—N1—H1B	115.0	C1—C6—H6	119.9
H1A—N1—H1B	111.2	O1—C7—N2	121.8 (2)
C7—N2—N3	119.3 (2)	O1—C7—C4	122.1 (2)
C7—N2—H2	120.4	N2—C7—C4	116.1 (2)
N3—N2—H2	120.4	N3—C8—C9	118.3 (2)
C8—N3—N2	115.1 (2)	N3—C8—H8	120.9
O3—N4—O2	123.5 (2)	C9—C8—H8	120.9
O3—N4—C10	118.12 (19)	C10—C9—C14	117.0 (2)
O2—N4—C10	118.3 (2)	C10—C9—C8	125.2 (2)
C2—C1—N1	119.9 (2)	C14—C9—C8	117.8 (2)
C2—C1—C6	118.8 (2)	C9—C10—C11	121.9 (2)
N1—C1—C6	121.0 (2)	C9—C10—N4	121.26 (19)
C3—C2—C1	120.0 (2)	C11—C10—N4	116.7 (2)
C3—C2—H2A	119.9	C12—C11—C10	119.5 (2)
C1—C2—H2A	119.9	C12—C11—H11	120.2
C2—C3—C4	121.2 (2)	C10—C11—H11	120.2
C2—C3—H3	119.4	C11—C12—C13	119.7 (2)
C4—C3—H3	119.4	C11—C12—H12	120.2
C5—C4—C3	118.2 (2)	C13—C12—H12	120.2
C5—C4—C7	119.0 (2)	C12—C13—C14	120.6 (2)
C3—C4—C7	122.6 (2)	C12—C13—H13	119.7
C6—C5—C4	121.0 (2)	C14—C13—H13	119.7
C6—C5—H5	119.4	C13—C14—C9	121.2 (2)
C4—C5—H5	119.4	C13—C14—H14	119.4
C5—C6—C1	120.2 (2)	C9—C14—H14	119.4
			
C7—N2—N3—C8	177.7 (2)	N3—C8—C9—C10	−152.8 (2)
N1—C1—C2—C3	−179.9 (2)	N3—C8—C9—C14	32.2 (3)
C6—C1—C2—C3	−1.3 (4)	C14—C9—C10—C11	2.8 (4)
C1—C2—C3—C4	0.9 (4)	C8—C9—C10—C11	−172.1 (2)
C2—C3—C4—C5	0.1 (4)	C14—C9—C10—N4	−176.2 (2)
C2—C3—C4—C7	−174.8 (2)	C8—C9—C10—N4	8.9 (4)
C3—C4—C5—C6	−0.8 (4)	O3—N4—C10—C9	−167.6 (2)
C7—C4—C5—C6	174.3 (2)	O2—N4—C10—C9	12.8 (4)
C4—C5—C6—C1	0.3 (4)	O3—N4—C10—C11	13.3 (3)
C2—C1—C6—C5	0.7 (4)	O2—N4—C10—C11	−166.2 (2)
N1—C1—C6—C5	179.2 (2)	C9—C10—C11—C12	−1.9 (4)
N3—N2—C7—O1	−6.0 (3)	N4—C10—C11—C12	177.2 (2)
N3—N2—C7—C4	170.59 (19)	C10—C11—C12—C13	−0.5 (4)
C5—C4—C7—O1	−22.4 (4)	C11—C12—C13—C14	1.8 (4)
C3—C4—C7—O1	152.4 (2)	C12—C13—C14—C9	−0.7 (4)
C5—C4—C7—N2	161.0 (2)	C10—C9—C14—C13	−1.6 (4)
C3—C4—C7—N2	−24.1 (3)	C8—C9—C14—C13	173.8 (2)
N2—N3—C8—C9	−175.25 (18)		

### Hydrogen-bond geometry (Å, º)

**Table:**

*D*—H···*A*	*D*—H	H···*A*	*D*···*A*	*D*—H···*A*
N1—H1*A*···N1^i^	0.89	2.44	3.287 (4)	162
N1—H1*B*···O1^ii^	0.89	2.35	3.164 (3)	153
N2—H2···O1^iii^	0.86	2.13	2.843 (3)	142
C2—H2*A*···O1^ii^	0.93	2.56	3.329 (3)	140

Symmetry codes: (i) −*x*+3, *y*+1/2, −*z*; (ii) *x*+1, *y*−1, *z*; (iii) *x*, *y*−1, *z*.
